# Effects of Combined Resistance and Power Training on Cognitive Function in Older Women: A Randomized Controlled Trial

**DOI:** 10.3390/ijerph17103435

**Published:** 2020-05-14

**Authors:** Hélio José Coelho-Júnior, Ivan de Oliveira Gonçalves, Ricardo Aurélio Carvalho Sampaio, Priscila Yukari Sewo Sampaio, Eduardo Lusa Cadore, Riccardo Calvani, Anna Picca, Mikel Izquierdo, Emanuele Marzetti, Marco Carlos Uchida

**Affiliations:** 1 Applied Kinesiology Laboratory–AKL, School of Physical Education, University of Campinas, Campinas 13083-851, SP, Brazil; sampaiorac@gmail.com (R.A.C.S.); pryukari@hotmail.com (P.Y.S.S.); uchida@fef.unicamp.br (M.C.U.); 2 Institute of Internal Medicine and Geriatrics, Università Cattolica del Sacro Cuore, 00168 Rome, Italy; riccardo.calvani@gmail.com (R.C.); anna.picca1@gmail.com (A.P.); 3 Rehabilitation unit, Mãe Mariana unit, Poá 08562-460, SP, Brazil; 4 Center of Health Sciences, University of Mogi das Cruzes, Mogi das Cruzes 08780-911, SP, Brazil; ivanogedfisica@gmail.com; 5 School of Physical Education, Physiotherapy and Dance, Federal University of Rio Grande do Sul, Porto Alegre 90040-060, RS, Brazil; edcadore@yahoo.com.br; 6 Fondazione Policlinico Universitario “Agostino Gemelli” IRCCS, 00168 Rome, Italy; 7 Navarrabiomed, Complejo Hospitalario de Navarra (CHN)-Universidad Pública de Navarra (UPNA), Navarra Institute for Health Research (IdiSNA), 31008 Pamplona, Spain; mikel.izquierdo@gmail.com; 8 GICAEDS Group, Faculty of Physical Culture, Sport and Recreation, Universidad Santo Tomás, Bogotá 7290, Colombia; 9 CIBER of Frailty and Healthy Aging (CIBERFES), Instituto de Salud Carlos III, 28220 Madrid, Spain

**Keywords:** exercise, high-speed resistance training, memory, dual-task, dementia, frailty

## Abstract

The present study compared the effects of traditional resistance training (TRT) and combined power training (PT) and TRT (PTRT) on cognitive parameters and serum brain-derived neurotrophic factor (BDNF) levels in non-demented, well-functioning, community-dwelling older women. Forty-five older women were randomized into one of three experimental groups: TRT, PTRT, and control group (CG). Cognitive tests explored global cognitive function, short-term memory, and dual-task performance. Serum BDNF levels were assessed at baseline and after the intervention. Exercise sessions were performed twice a week over 22 weeks. In TRT, exercise sessions were based on three sets of 8–10 repetitions at “difficult” intensity. In PTRT, the first session was based on PT (three sets of 8−10 repetitions at “moderate” intensity), while the second session was similar to the TRT. Our analyses indicated that overall cognitive function, short-term memory, and dual-task performance were similarly improved after TRT and PTRT. Serum BDNF concentrations were not altered by any training protocol. In conclusion, the two RT programs tested in the present trial improved global cognitive function, short-term memory and dual task performance in non-demented, well-functioning, community-dwelling older women. In addition, our findings suggest that mechanisms other than BDNF may be associated with such improvements.

## 1. Introduction

Cognition refers to the mental process responsible for the interaction between the mind and the world [1]. Conceptually, cognition involves crystallized abilities—the ability to use learned knowledge and experiences—and fluid abilities—the ability to use logic in new situations, solve new problems, and identify patterns [2]. These abilities are differently affected with aging, such that crystallized abilities commonly remain stable from age 60 until age 80, while fluid abilities decline after 60 years [2]. These factors have important clinical applications, since reductions in fluid abilities are strongly associated with the development of age-related syndromes (e.g., frailty) [3,4] and diseases (e.g., Alzheimer’s disease [AD]) [5].

Among the cognitive domains affected by aging, short-term memory and dual-task performance have gained widespread attention owing to their relevance to the individual’s autonomy. In fact, short-term memory — the capacity of holding an amount of information for a short period of time [6] — is involved in various aspects of cognition (e.g., executive function) and every-day activities, such as conversation, reading, learning, mathematics [6,7]. Furthermore, a progressive decline in this cognitive domain is commonly found in AD patients [8].

On the other hand, dual-task, which involves the simultaneous performance of motor and cognitive tasks, challenges many cognitive domains, including attention, inhibitory control, and executive function [9,10]. In recent years, dual-task performance has been associated not only with physical outcomes (e.g., falls, disability) [11], but also with biological markers of dementia [9] and cognitive status [10,12], suggesting that reduced dual-task performance may serve as a marker of cognitive decline.

In addition to cognitive changes that occur late in life, older adults are highly susceptible to experience depressive symptoms (DS) [13]. Depression refers to a mood disorder characterized by the presence of multiple symptoms, including insomnia or hypersomnia, weight loss or gain, changes in appetite, psychomotor agitation or retardation, fatigue, and recurrent thoughts of death and suicidal ideation [14]. As depression progress, individuals show reduced quality of life, physical performance, and mental functioning, as well as increased disability [15]. Although depression is a treatable condition, its management is commonly based on antidepressants and electroconvulsive therapy [14].

According to the World Health Organization (WHO) [15], the maintenance and, possibly, improvement of mental health should be prioritized by health professionals caring for older people to avoid the negative outcomes associated with dementia. To this aim, physical exercise has been proposed as a possible non-pharmacological intervention [16,17]. Nevertheless, solid evidence on the subject is still missing, which precludes the development of clinical guidelines.

Although there are several types of physical exercises, traditional resistance training (TRT), a type of exercise in which muscles work or hold against an applied force, is proposed as a first line therapy to counteract age-related neuromuscular decline [18,19]. Notably, increasing evidence indicates that TRT may also improve cognitive function [20,21,22,23] and DS [24] in older adults. Nevertheless, these findings were not confirmed in other studies [25,26], suggesting that more evidence is still needed.

Power training (PT), a type of resistance training (RT) in which concentric muscle contractions are performed as fast as possible at light-to-moderate loads [27], is recommended as part of RT programs for adults [18,28,29], based on the proposition that some aspects of physical function (e.g., chair rise, stair climb) may be more dependent on muscle power than on muscle strength [30,31,32].

However, only a few studies have investigated the impact of PT on cognitive function [33,34,35]. Existing studies were based on non-randomized designs (e.g., quasi-experimental) [33], short intervention periods (~12 weeks), or samples composed of young to middle aged adults [33,35] with mild-cognitive impairment [34] or dementia [33], limiting the extrapolation of findings to non-demented older adults.

Many mechanisms may mediate the effects of RT on cognitive function [22,36]. In the last years, a theoretical basis was created to support the role of myokines on physical exercise-induced cognitive improvements [37]. Myokines are molecules, cytokines or signaling peptides with pluripotent effects synthetized by contracting muscles [37,38,39,40]. According to proteomic studies, approximately 60 myokines are regulated in response to muscle contractions [41], although some of these molecules may be exclusively secreted by type II muscle fibers [42]. Notably, PT might stimulate the recruitment of type II muscle fibers to a comparable degree to TRT [43,44], which suggests that both RT protocols may similarly increase the synthesis of myokines with a role in cognitive functioning.

Brain-derived neurotrophic factor (BDNF) is one of the most studied myokines [37]. BDNF is involved in neurogenesis, synaptic plasticity, neuronal morphology, and neuropathology [45,46]. Nevertheless, despite the increased BDNF expression in rats submitted to exercise training [47], many reports [48,49,50,51] have shown no changes in systemic BDNF levels in older adults after RT. A possible explanation for this fact is that investigations were based on short intervention periods (e.g., 6 weeks) and participants were patients with psychiatric diseases [48,49,50].

Alternatively, PT can improve cognitive function in older adults by increasing aerobic power [35]. According to Cherup et al. [35], increased aerobic capacity with PT may modify neural structures and stimulate vascular branching, contributing to improvements in neuronal function. This data suggest that the combination of these types of exercise training may elicit similar or greater changes in cognition than TRT alone, while older individuals are submitted to low cardiovascular and osteoarticular stresses [52,53,54]. However, no prior evidence investigated the effects of combined PT and TRT (PTRT) on cognitive function of older adults.

To fill this gap in knowledge, the present study was undertaken to verify whether PTRT may promote similar or greater benefits in cognitive function and DS in physically active non-demented community-dwelling older women. In addition, BDNF was investigated as a possible underlying mechanism mediating the effects of RT on these parameters.

## 2. Materials and Methods

### 2.1. Study Design and Participants

This is a subgroup analysis of a randomized clinical trial [54] based on a priori hypothesis that TRT and PTRT could similarly improve cognitive function in community-dwelling older women. The main study used body composition and physical function as main outcomes [54]. Sample size calculation was based on the mean differences in Timed “Up-and-Go” (TUG) performance among three groups in four repeated measures [54]. All researchers, including assessors, exercise supervisors, and those responsible for statistical analysis knew where the participants were allocated. The current investigation was carried out over a total of 26 weeks, with the first and the last two weeks dedicated exclusively to evaluations. Cognitive function was also assessed at weeks 5 and 14. RT protocols occurred over 22 weeks. Ethics approval was granted by the Human Research Ethics Committee of the University of Campinas (Campinas - SP, Brazil; Protocol No. 835.733). All participants provided written informed consent prior to inclusion. All study procedures were conducted following the principles of the Declaration of Helsinki. The study was registered as a clinical trial in Clinicaltrial.gov (identifier: NCT03443375).

Women aged ≥60 years were recruited through advertisements from the Center for Older Adults of the city of Poá, SP, Brazil. Participants were eligible for inclusion if they: a) lived in the community; b) had no disability in basic and instrumental activities of daily living, based on Katz [55] and Pfeffer [56] indexes, respectively; c) were post-menopausal for at least one year; and d) had a physician authorization to participate in the trial. Exclusion criteria included having participated in a structured physical exercise training program in the past six months, engagement in other physical exercise programs during the study, missed four or more exercise sessions in a recurrent and sequential manner according to the records, presence of comorbidities associated with greater risk of falls (e.g., overall weakness, balance problems), self-reported falls in the previous six months, self-reported malnutrition, self-reported illiteracy, history of smoking or alcohol abuse in the prior five years, prescription of hormone replacement therapy and/or psychotropic drugs, and dementia according to the Mini-Mental Mental State Examination (MMSE) score adjusted by educational level [57,58]. We also excluded individuals who showed a clinical diagnosis of neurological and/or psychiatric, cardiovascular, pulmonary, and metabolic diseases, and skeletal muscle disorders.

Concealed randomized allocation into one of three experimental groups —TRT, PTRT, and control group (CG) — was performed by an independent researcher before baseline evaluations using a simple computer-generated list of random numbers.

### 2.2. Outcomes

The main outcome of the present study was cognitive function. Cognitive tests included the MMSE (i.e., global cognition), TUG with a cognitive task (TUG-cog) (i.e., dual-task), and picture memory test (i.e., short-term memory). Secondary outcomes included DS (i.e., 15-item Geriatric Depression Scale [GDS]) [59] and serum levels of BDNF. Cognitive tests and DS were assessed at baseline and at weeks 5, 14, and 23, while BDNF was measured at baseline and at week 23.

#### 2.2.1. Cognitive Function

All cognitive tests were administered face-to-face in a private silent room by a trained researcher.

##### Mini-Mental State Examination

A paper-pencil version of the MMSE was used to assess overall cognitive function. MMSE evaluates language, concentration, attention, short-term recall, visuospatial abilities, registration, and orientation through 11 questions. A total score ranging from 0 to 30 points is generated at the end of the test, with higher scores corresponding to better cognitive function [57,58].

##### TUG with a Cognitive Task

TUG-cog involves performing conventional TUG with a verbal fluency task (animal category). On the word “go”, participants named out loud as many animals they could, while they got up from a chair (total height: 87 cm; seat height: 45 cm; width: 33 cm;), walked three meters around a marker placed on the floor, came back to the same position, and sat back on the chair [12].

##### Picture Memory Test

The picture memory test is a paper-pencil test based on the analysis and memorization of an image, requiring attention and short-term memory. The test consists in a picture of a landscape on the beach with 41 objects commonly observed in daily life. Participants were instructed to study the picture for 1 min and memorize as many items as they could. After this encoding period, they were distracted for 5 min, and then asked to recall the objects in the picture. No time limitation was set for completing the test [60].

#### 2.2.2. Secondary Outcomes

DS were quantified using a short version of the GDS. The scale is composed of 15 questions about the feelings, or the frequency of the feelings, the person faced with certain conditions in life. The answers are based on a binary code: yes or no [59]. Serum BDNF concentrations were measured with an enzyme-linked immunosorbent assay using an ELISA kit (Sigma-Aldrich, Poole, UK; Ref: RAB0026). Briefly, serum samples were diluted 20-fold in the supplied assay diluent and measured against a standard curve with BDNF concentrations ranging from 62.5 pg/mL to 4000 pg/mL. Serum samples and BDNF standards were incubated for 2 h on the captured anti-human BDNF coated microplate. Then, a monoclonal antibody specific for human BDNF conjugated to horseradish peroxidase was added to the wells. After a wash step, the supplied tetramethylbenzidine (TMB) substrate solution was added to the wells and a blue color developed in proportion to the amount of human BDNF present in the samples. Color development was stopped with 2 N sulfuric acid, turning the color in the wells to yellow. The absorbance was measured on a spectrophotometric microplate reader (Bio-Rad, Hercules, CA, USA). All samples were tested in duplicates. Sample BDNF concentrations were determined by non-linear regression from the standard curves [61].

### 2.3. Resistance Training Program

Both resistance training protocols were carried out over 26 weeks. Exercise sessions were conducted in pairs, for approximately one hour, under the supervision of fitness instructors in the exercise room of the Center for Older Adults of the city of Poá, SP, Brazil. The first and the last two weeks were dedicated to evaluations. RT sessions were scheduled as two non-consecutive sessions weekly for 24 weeks.

During weeks 1−4, participants performed a familiarization period based on 12−15 submaximal repetitions at an “easy” intensity according to the Rating of Perceived Exertion (RPE, CR−10) adapted Borg scale [62] in nine exercises for major muscle groups. The number of sets was progressively increased from two, in the first two weeks, to three, in the 3rd and 4^th^ weeks. The main RT period was the same for TRT and PTRT. RT programs were equalized for frequency, sets, repetitions, and rest interval, but not exercise intensity and the velocity of concentric muscle contraction. In this period, participants performed three sets of 8–10 submaximal repetitions with a 1-min rest interval period between sets in the full range of motion. A brief warm-up was provided at the beginning of each session.

Exercise intensity and the velocity of concentric muscle contractions were modified differently for each group according to the peculiarities of each type of RT [18]. For the TRT, participants were instructed to perform exercise sessions at a “difficult” intensity (RPE = 5−6) [62] using exercise machines (Johnson Health Tech, Taichung, Taiwan) and free weights. The concentric and eccentric phases were carried out for 2 s. The list of exercises performed by TRT was: 1st) seated row, 2nd) leg press, 3rd) chest press, 4th) seated leg curl, 5th) lateral arm raise, 6th) calf raise, 7th) arm curl, 8th) triceps pushdown, and 9th) abdominal crunch.

For PTRT, the first week session was the same as for TRT, while the second week session was performed at a “moderate” intensity (RPE = 3) [62] using elastic bands (progressive order of intensity [color], yellow, red, green, and blue) (TheraBand, Akron, OH, USA). Participants were encouraged to perform concentric contractions as quickly as possible. A researcher was responsible for supervising participants for proper velocity. A regenerative week based on three sets of 12–15 submaximal repetitions of each exercise, at “easy” intensity (RPE = 2) [62] was performed every four weeks. The list of exercises used during the second week session in PTRT was: 1st) squat on the chair (until 90° knee flexion), 2nd) chest press, 3rd) seated leg curl, 4th) seated row, 5th) frontal arm raise, 6th) calf raise, 7th) arm curl, 8th) triceps pushdown, and 9th) abdominal crunch.

The training load was adjusted based on the RPE method, using the CR−10 scale [62]. After each set, participants were shown the scale and asked: “How would you rate your effort?” [63]. If an RPE below the expectation was reported, the weight was increased by 2–5% for upper extremity exercises and by 5–10% for lower extremity exercises [18].

### 2.4. Control Group

Participants in the CG did not receive exercise intervention and were contacted every 15 days to ensure that they were adhering to the study protocol.

### 2.5. Statistical Analyses

Data are shown as mean ± standard deviation (SD). Normality of data was tested using the Kolmogorov–Smirnov test. Baseline comparisons among groups were performed using one-way analysis of variance (ANOVA) followed by Tukey’s post-hoc test as appropriate. Greenhouse–Geisser corrections were applied for data that violated sphericity assumptions. A group × time repeated-measures ANOVA followed by Dunnet post-hoc test was performed to assess differences among different times of evaluations and treatments. Relative percentage of change (∆%) was calculated according to the formula:

(23rd week−Baseline*100)/Baseline

Cohen’s effect size (ES) and ∆% were calculated based on baseline−23rd week changes. ES was calculated according to the following formula:

Baseline−23rd week/(√[(SD1² + SD2²)/2])

ES was classified as small (0.15−0.39), medium (0.40−074), and large (≥0.75) according to Cohen’s *d* [64]. The level of significance was set at alpha = 5% (*p* < 0.05). All analyses were performed using the GraphPad Prism 6.0. (GraphPad Software, San Diego, CA, USA).

## 3. Results

**Figure 1:**
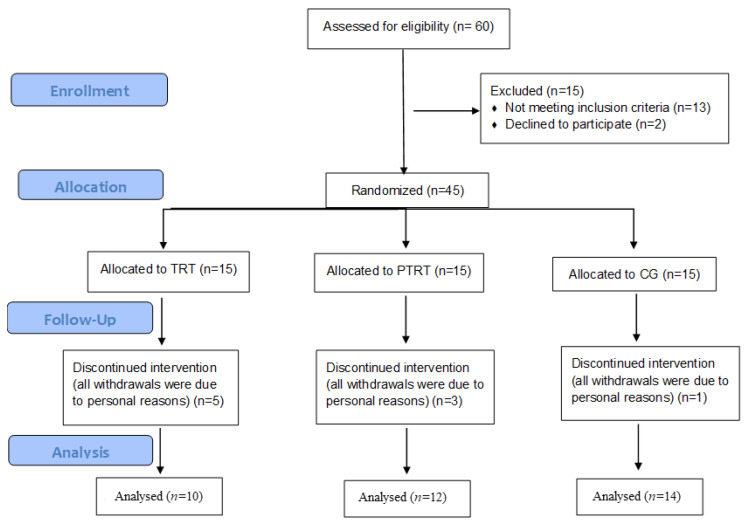
Flowchart of the study. TRT = Traditional resistance training; PTRT = Power training resistance training; CG = Control group.

One-hundred and three older women were recruited for the present study and 60 were assessed for eligibility. Of these, six had a clinical diagnosis of type II diabetes, four had a previous myocardial infarction, three reported at least one fall event in the previous year, and two declined to participate, leaving 45 participants, who were randomly allocated into TRT, PTRT, and CG. Over the follow-up period, nine participants withdrew from the trial, five from the TRT, three from the PTRT, and one from the CG. All withdrawals were due to personal reasons (Figure 1). Dropouts had lower TUG-cog performance in steps-domain (21.4 ± 1.5; *p* = 0.0001) and Katz index (5.2 ± 0.3; *p* = 0.01), as well as higher Pfeffer index (1.7 ± 0.4; *p* = 0.01) and MMSE (25.0 ± 0.2; *p* < 0.00001). RT adherence was 88.7% for TRT and 90.0% for PTRT.

### 3.1. Baseline Characteristics

Table 1 lists the main characteristics of study participants at baseline according to group allocation. There were no differences among groups for general characteristics or cognitive function. However, total training workload (total number of sets × total number of repetitions × total weight lifted [kg]) was higher in TRT (172.1 ± 8.6 kg) than that in PTRT (103.2 ± 3.2 kg) (*p* < 0.001).

### 3.2. Effects of TRT and PTRT on Cognitive Function

Table 2 indicates the effects of time, group, and their interaction on cognitive function. There was a significant effect of time on MMSE and TUG-cog (time-domain), group on TUG-cog (time and steps), and interaction for all parameters. Both TRT and PTRT produced significant improvements in global cognitive function (MMSE), short-term memory (i.e., picture memory test), and dual-task performance (time and steps). However, improvements in MMSE after TRT were only significant in relation to CG, but not in comparison to baseline. A similar pattern was observed for short-term memory in PTRT. A large ES was attributed to changes in global cognitive function and dual-task in TRT and PTRT, and for short-term memory in TRT.

### 3.3. Time-Course Effects of TRT and PTRT on Cognitive Function

**Figure 2:**
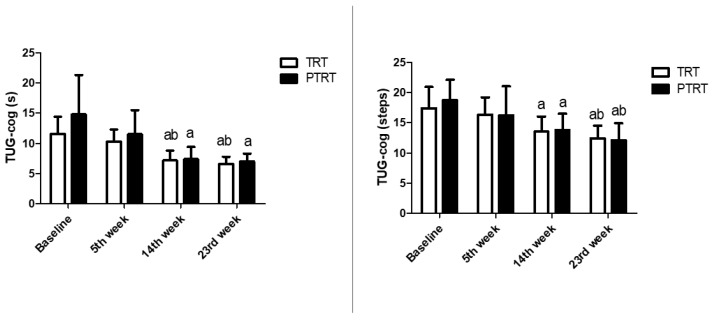
Time-course effects of TRT and PTRT on cognitive function. TRT = Traditional resistance training; PTRT = Power training resistance training; TUG-cog = Timed “Up-and-Go”- cognitive; ^a^ *p* < 0.05 vs. Baseline; ^b^ *p* < 0.05 vs. 5th week.

Time-course analysis of TRT and PTRT are shown in Figure 2. Significant improvements in dual-task domains were already detectable at week 14 in TRT and PTRT (time *p* ≤ 0.001; steps *p* ≤ 0.01 for both). Results indicated that time-domain and steps-domain were significantly improved at weeks 14 and 23 in relation to week 5 and baseline.

### 3.4. Effects of TRT and PTRT on Depressive Symptoms

Table 3 shows the effects of time, group, and their interaction on DS. There was a significant effect of time (*p* = 0.01), but no treatment (*p* > 0.05) or interaction (*p* > 0.05) on DS. GDS scores were not altered by any training protocol. However, non-significant changes in GDS in TRT were accompanied by a large ES classification.

### 3.5. Effects of TRT and PTRT on BDNF Levels

**Figure 3:**
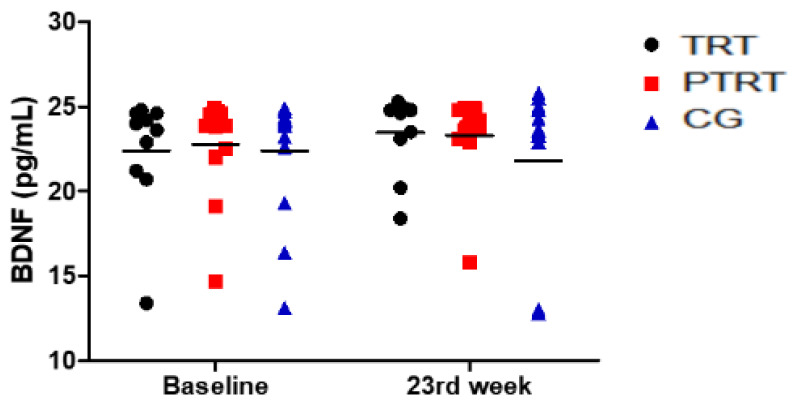
Effects of TRT and PTRT on BDNF levels. TRT = Traditional resistance training; PTRT = Combined power training and resistance training; CG = Control group.

Serum BDNF levels are shown in Figure 3. No intra- (*p* = 0.81; TRT: −0.25, Small; PTRT = −0.18, Small; CG = 0.14, Unclassifiable) or inter-group differences in serum BDNF concentrations were observed.

## 4. Discussion

Findings of the present study indicate that RT, both traditional and combined with PT, improved global cognitive function, short-term memory, and dual-task performance in community-dwelling older women. In contrast, no significant changes were observed in GDS scores or serum BDNF concentrations following either RT regimens. Remarkably, TRT and PTRT elicited greater improvements in MMSE (3.8 and 3.4, respectively) than the minimal clinically important difference (MCID) for the test (i.e., 3.0) [65]. Our results also indicate that cognitive benefits elicited by RT may be time-dependent. Indeed, improvements in dual-task performance were already significant after 14 weeks, while changes in short-term memory and global cognitive function reached statistical significance at week 23.

These findings add to and expand the existing knowledge by suggesting that the velocity of concentric muscle contractions has a role in cognitive responses to RT. In fact, TRT and PTRT similarly improved overall cognitive function (16.2% and 14.4%), short-term memory (56.1% and 39.3%), and dual-task performance (43.1% and 52.7%) in non-demented, well-functioning, older women, despite significant differences in total workloads between groups.

A likely explanation for our findings is that explosive concentric muscle contractions performed at moderate loads might stimulate the recruitment of type II muscle fibers to a similar degree than normal-velocity concentric muscle contractions performed at moderate-to-high loads [43,44]. Although this model has been widely used to explain similar neuromuscular improvements observed after both PT and TRT [66,67], our findings raised the hypothesis that this phenomenon might also potentially stimulate cognitive enhancements.

We initially assumed that the stimulation of type II muscle fibers by both TRT and PTRT could promote the synthesis and release of myokines [42], molecules with autocrine, paracrine, and endocrine effects on numerous metabolic process, including energy expenditure and lipid (e.g., lipolysis, adipocyte browning, fat-free acids oxidation), muscular (e.g., glucose uptake) and liver metabolism (e.g., glycogenolysis and glycogenesis) [68,69,70,71,72]. More recently, a theoretical basis has been proposed to support the role of myokines in physical exercise-induced cognitive improvements [37].

Hence, BDNF was investigated as a possible signaling molecule involved in the cognitive adaptations induced by our RT protocols. However, serum BDNF levels were unchanged by either TRT or PTRT. Our results are in keeping with those of previous studies in which BDNF levels were also unaffected by various RT protocols [48,49,50]. It may be hypothesized that factors other than RT prescription, such as specific comorbidities, lifestyle habits, and even genetic variations may be associated with changes in systemic BDNF levels after RT [73].

One of the main limitations of the present study is the lack of assessment of other myokines that may contribute to exercise-induced improvements in cognitive function [37]. In particular, insulin-like growth factor 1 (IGF-1) is acutely [74] and chronically [20] increased in response to RT in older adults and systemic IGF-1 levels are significantly associated with hippocampal perfusion and volume [75]. In addition, IGF-1 is expressed in brain areas strongly associated with memory formation (e.g., hippocampus, cortex) [76] and seems to be critically involved in exercise-induced improvements in neuronal activation and cell proliferation [76,77].

Functional and structural brain changes have also been associated with cognitive improvements in older adults after RT. Hong et al. [25] observed changes in electroencephalogram patterns in healthy older adults after a 12-week elastic band RT program. According to researchers, such changes in brain activity may reflect a resistance exercise-induced increase in blood flow, neurotransmitters, and mitochondrial metabolism. Regarding brain morphology, Liu-Ambrose et al. [21] found reduced whole-brain volume parallel to improvements in executive function of older adults who performed RT with different frequencies. The authors argued that reduced whole-brain volume in response to RT may indicate decreased brain β-amyloid content. Hence, more studies are needed to provide further insight into factors influencing the effects of RT on cognitive function.

The present findings are supported by previous studies that found improved cognitive capacity in response to different RT programs. In a seminal study, Cassilhas et al. [20] observed increased memory performance and verbal concept formation in older adults who had undergone moderate (50% 1RM) or high-intensity RT (80% 1RM). In addition, Liu-Ambrose [21] demonstrated that RT performed once or twice a week similarly improved executive functions (i.e., selective attention, conflict resolution) in community-dwelling older women. As a whole, our results and findings from previous studies suggest that cognitive adaptations in response to RT occur regardless of the type, intensity, and frequency of RT.

Another important finding of the present study was that cognitive adaptations in response to RT seemed to be time-dependent, since significant improvements in dual task performance were detectable at week 14, while improvements in short-term memory were detectable only later (i.e., 23rd week). Interestingly, prior investigations did not observe significant changes in cognitive parameters after short-term RT (3 months) [26]. On the other hand, our findings are supported by previous studies that found improved memory and executive function after longer interventions (~6 months) [20,21]. Hence, a minimum duration of intervention (≥ 3 months) seems to be necessary to elicit significant changes in some cognitive parameters [78].

The current findings may have important clinical implications. Indeed, overall cognitive function measures (e.g., MMSE) are routinely used in clinics and research for cognitive [79,80,81] and low scores in these tests are associated with numerous negative health-related outcomes, such as insomnia, loneliness, and dementia [82,83,84,85]. Improvements in short-term memory may have a direct impact on an individual’s quality of life, given its close association with various aspects of cognition and every-day activities, such as conversation, reading, learning, mathematics [6,7]. In addition, reduced short-term memory is commonly found in people with AD [8]. Finally, reduced dual-task performance is observed according to dementia severity [10,12], and older adults with impaired ability to perform both motor and cognitive tasks simultaneously are at higher risk of falls and death [11].

As a practical application of our findings, health professionals may find cognitive benefits after both TRT and PTRT. Nevertheless, greater neuromuscular improvements were found in TRT in comparison to PTRT [54] and, therefore, this design of RT should be prioritized to maintain or improve cognitive function and physical performance in healthy community-dwelling older women. Alternatively, PTRT may be indicated for older women who unable to go to the gym twice a week or prefer to exercise outdoors without dumbbells or machines.

Although reduced DS was observed after both TRT and PTRT, with a *large* ES being attributed to TRT, no significant changes were observed following either RT regimen. A possible explanation for these findings may be that larger reductions in DS after RT are achievable in participants with baseline GDS scores indicative of mild to moderate depression [24]. In this respect, despite the high heterogeneity in GDS scores in our sample (0−10 points), the mean score was not suggestive of DS [59].

The present study is not free of limitations. First, we investigated a small sample size composed exclusively of older women. Second, the lack of a comprehensive neuropyschological assessment impeded the appreciation of the effects of TRT and PTRT on other cognitive domains. Third, the hypothesis that the learning effect influenced our findings cannot been ruled out, given that MMSE has a single format that was applied repeatedly over the study. Fourth, a different number of measurements were performed by experimental and control groups. Fifth, the use of brain flow measures could contribute to understanding the impact of RT protocols on cerebrovascular function. In addition, the low GDS scores of participants in the present study does not allow inference on the impact of RT on DS in older adults with higher scores. Finally, the study is a subgroup analysis of a randomized clinical trial [54]. One of the main concerns with this type of analysis is in regard to the sample size, given that the power calculation was based on variables other than those investigated in the secondary analysis [85]. To understand the limitations of our sample size, we performed a post-hoc power calculation conducted from ANOVA based on an ES of 1.2 for changes in overall cognitive function [86], power of 0.8 and an alpha set at 0.05. Results indicated that fifty-seven participants (*n*= 19 per group) would be necessary at baseline to ensure adequate precision around the estimates generated. Hence, our findings must be confirmed in future randomized clinical trials using cognitive function as the primary outcome.

## 5. Conclusions

The two RT programs tested in the present trial improved global cognitive function, short-term memory, and dual task performance in non-demented, well-functioning, community-dwelling older women. In addition, our findings suggest that cognitive adaptations in response to RT may be time-dependent, such that improvements in dual-task performance are detectable earlier than those in short-term memory and global cognitive function.

## Figures and Tables

**Table 1:** Baseline characteristics according to group allocation.

Variables	TRT (*n* = 10)	PTRT (*n* = 12)	CG (*n* = 14)	*p*-Value
**General Characteristics**				
Age (years)	67.0 ± 6.2	66.7 ± 5.1	66.7 ± 4.6	0.98
Weight (kg)	71.7 ± 12.9	68.2 ± 12.2	69.3 ± 20.4	0.61
Height (cm)	153 ± 0.0	158 ± 0.0	160 ± 0.1	0.18
Body Mass Index (kg/m²)	30.2 ± 4.1	27.8 ± 6.2	27.0 ± 7.7	0.15
Katz Index (points)	5.8 ± 0.7	5.5 ± 0.6	5.2 ± 0.4	0.16
Pfeffer Index (points)	0.4 ± 0.6	1.2 ± 1.4	2.0 ± 2.6	0.11
Schooling (years)	7.6 ± 4.1	7.4 ± 4.4	8.5 ± 4.3	0.79
GDS (points)	3.3 ± 2.1	3.3 ± 3.2	2.2 ± 2.4	0.87
Total Workload (kg) *	172.1 ± 8.6	103.2 ± 3.2	NA	0.001
**Cognitive Domains**				
MMSE (points)	23.4 ± 4.5	23.6 ± 2.4	24.8 ± 3.0	0.51
TUG-cog (s)	11.6 ± 2.8	14.8 ± 6.5	11.9 ± 2.7	0.16
TUG-cog (steps)	17.4 ± 3.5	18.7 ± 3.4	18.5 ± 4.1	0.65
Short-term Memory Test (points)	8.2 ± 4.4	8.4 ± 4.4	8.1 ± 2.9	0.98

Data are shown as mean ± SD. TRT = Traditional resistance training; PTRT = Combined power training and resistance training; CG = Control group; GDS = Geriatric depressive scale; TUG-cog = Timed “Up-and-Go” with a cognitive task; NA= Not applicable. * Independent T-test.

**Table 2:** Cognitive function at baseline and after 5, 14 and 23 weeks according to group allocation.

Time Points	TRT (*n* = 10)	PTRT (*n* = 12)	CG (*n* = 14)	*p*-Value		
**MMSE (points)**				**Time**	**Group**	**Time × Group**
Baseline	23.4 ± 4.5 (15−30)	23.6 ± 2.4 (18−27)	24.8 ± 3.0 (19−30)			
23rd week	27.2 ± 4.1 (18−30) ^b^	27.0 ± 2.5 (23−30) ^a,b^	23.2 ± 3.1 (18−28)			
ES (classification)	−1.0 (large)	−2.0 (large)	0.33 (small)			
∆%	16.2	14.4	−6.5	0.001	>0.05	0.01
**TUG-cog (s)**						
Baseline	11.6 ± 2.8 (7−18)	14.8 ± 6.5 (8−30)	11.9 ± 2.7 (8.6−16.5)			
23rd week	6.6 ± 1.2 (5−8.6) ^a,b^	7.0 ± 1.3 (5.4−9) ^a,b^	12.3 ± 2.3 (9.0−15.6)			
ES (classification)	3.16 (large)	1.62 (large)	−0.50 (medium)			
∆%	−43.1	−52.7	3.4	0.001	0.01	0.001
**TUG-cog (steps)**						
Baseline	17.4 ± 3.5 (14−24)	18.7 ± 3.4 (14−24)	18.6 ± 3.6 (14−25)			
23rd week	12.4 ± 2.1 (9−17) ^a,b^	12.1 ± 2.8 (5.6−17) ^a,b^	16.5 ± 3.2 (13−24)			
ES (classification)	1.96 (large)	2.35 (large)	0.66 (medium)			
∆%	−28.7	−35.3	−11.3	>0.05	0.01	0.001
**Short-term Memory Test (points)**						
Baseline	8.2 ± 4.4 (1−14)	8.4 ± 4.4 (0−14)	8.1 ± 2.9 (4−12)			
23rd week	12.8 ± 5.2 (4−20) ^a^^,^^b^	11.7 ± 6.2 (3−22) ^b^	7.0 ± 2.7 (2−10)			
ES (classification)	−0.95 (large)	−0.61 (medium)	0.39 (small)			
∆%	56.1	39.3	−13.6	>0.05	>0.05	0.01

Data are shown as mean ± SD. ∆% = Relative percentage of change; TRT = Traditional resistance training; PTRT = Combined power training and resistance training; CG = Control group; MMSE = Mini-mental state examination; ES = Effect size; TUG-cog = Timed “Up-and-Go” with a cognitive task. ^a^ *p* < 0.05 vs. Baseline; ^b^ *p* < 0.05 vs. CG.

**Table 3:** Prevalence of depressive symptoms at baseline and after 5, 14 and 23 weeks according to group allocation.

Time Points	TRT (*n* = 10)	PTRT (*n* = 12)	CG (*n* = 14)	*p*-Value		
		GDS (points)		Time	Group	Time × Group
Baseline	3.3 ± 2.1 (1.0−8.0)	3.3 ± 3.2 (0.0−10.0)	3.0 ± 3.1 (0.0−9.0)			
23rd Week	0.9 ± 1.8 (0.0−6.0)	1.9 ± 1.8 (0.0−6.0)	2.5 ± 2.5 (0.0−9.0)			
ES (23rd vs. Baseline)	1.17 (large)	0.53 (medium)	0.17 (small)			
∆%	−72.2	−42.4	−16.6	0.01	>0.05	>0.05

Data are shown as mean ± SD. ∆% = Relative percentage of change; TRT = Traditional resistance training; PTRT = Combined power training and resistance training; CG = Control group; ES = Effect size; GDS = Geriatric depressive scale.
